# Dissection of the impact of prioritized QTL-linked and -unlinked SNP markers on the accuracy of genomic selection^1^

**DOI:** 10.1186/s12863-021-00979-y

**Published:** 2021-08-11

**Authors:** Ashley S. Ling, El Hamidi Hay, Samuel E. Aggrey, Romdhane Rekaya

**Affiliations:** 1 Department of Animal and Dairy Science, The University of Georgia, 30602 Athens, GA USA; 2 USDA Agricultural Research Service, Fort Keogh Livestock and Range Research Laboratory, Miles City, MT 59301 USA; 3 Department of Poultry Science, The University of Georgia, 30602 Athens, GA USA; 4 Institute of Bioinformatics, The University of Georgia, 30602 Athens, GA USA; 5 Department of Statistics, The University of Georgia , 30602 Athens, GA USA

**Keywords:** F_ST_ scores, Marker preselection, Genomic prediction, Accuracy

## Abstract

**Background:**

Use of genomic information has resulted in an undeniable improvement in prediction accuracies and an increase in genetic gain in animal and plant genetic selection programs in spite of oversimplified assumptions about the true biological processes. Even for complex traits, a large portion of markers do not segregate with or effectively track genomic regions contributing to trait variation; yet it is not clear how genomic prediction accuracies are impacted by such potentially nonrelevant markers. In this study, a simulation was carried out to evaluate genomic predictions in the presence of markers unlinked with trait-relevant QTL. Further, we compared the ability of the population statistic F_ST_ and absolute estimated marker effect as preselection statistics to discriminate between linked and unlinked markers and the corresponding impact on accuracy.

**Results:**

We found that the accuracy of genomic predictions decreased as the proportion of unlinked markers used to calculate the genomic relationships increased. Using all, only linked, and only unlinked marker sets yielded prediction accuracies of 0.62, 0.89, and 0.22, respectively. Furthermore, it was found that prediction accuracies are severely impacted by unlinked markers with large spurious associations. F_ST_-preselected marker sets of 10 k and larger yielded accuracies 8.97 to 17.91% higher than those achieved using preselection by absolute estimated marker effects, despite selecting 5.1 to 37.7% more unlinked markers and explaining 2.4 to 5.0% less of the genetic variance. This was attributed to false positives selected by absolute estimated marker effects having a larger spurious association with the trait of interest and more negative impact on predictions. The Pearson correlation between F_ST_ scores and absolute estimated marker effects was 0.77 and 0.27 among only linked and only unlinked markers, respectively. The sensitivity of F_ST_ scores to detect truly linked markers is comparable to absolute estimated marker effects but the consistency between the two statistics regarding false positives is weak.

**Conclusion:**

Identification and exclusion of markers that have little to no relevance to the trait of interest may significantly increase genomic prediction accuracies. The population statistic F_ST_ presents an efficient and effective tool for preselection of trait-relevant markers.

## Background

Whole-genome marker information has been successfully utilized through genomic selection (GS) in many livestock and plant genetic improvement programs for the prediction of genomic merit and has led to a significant increase in the rate of genetic gain in these species [1]. This has been partly a result of increased prediction accuracy for selection candidates, particularly for individuals with no phenotypes or progeny of their own [2]. Such improvement in accuracy is due to a better modeling of the Mendelian sampling (MS) using genomic information compared to using only pedigree information.

Though millions of single nucleotide polymorphisms (SNPs) have been discovered in human [3], livestock [4], and plant [5] genomes, relatively high accuracies have been achieved using marker panels that utilize just a fraction of these markers [6, 7]. The falling costs of full genome sequencing and genotyping combined with more reference genomes and the availability of imputation algorithms have now allowed the regular use of high-density and sequence genotypes in genomic analyses.

It has been suggested that sequence data has the potential to significantly improve the accuracy of genomic predictions by increasing the linkage disequilibrium (LD) between quantitative trait loci (QTL) and SNPs or even making available the genotypes of causal loci [8–10]. Early simulation studies found optimistic potential for the use of sequence data in GS. Meuwissen and Goddard [9] estimated that accuracies could be improved by more than 40% when using sequence data compared to low-density SNP panels, but concluded that this was likely due to the weak relationship structure of the training population and did not expect the same results in real livestock populations due to the long-ranging LD and strong family structures. Druet et al. [10] found that accuracies could be increased by up to 28% using sequence data compared to the equivalent of a bovine 50 k SNP chip when the trait was controlled by rare QTL; however, these gains were largely lost when the sequence genotypes were imputed, likely as a result of lower imputation accuracy of rare markers that would be most effective in tracking causal loci with low minor allele frequencies.

Most results from real data have found little to no improvement in accuracy using high-density and sequence data for genomic prediction [11–14]. This lack of improvement has in some cases been attributed to the fact that low- and moderate-density panels are sufficient to capture realized additive relationships across the whole genome. Furthermore, a marginal decline in accuracy with the increase in SNP density was observed in some cases [12, 14], which results in part from overparameterization of the model [15]. This is not a surprising occurrence, as a disproportional increase in the number of unknown parameters in the association model relative to the number of observations available in the training set will lead to the well-known small *n* large *p* problem.

Models that intrinsically perform variable selection (e.g., BayesB, LASSO, and elastic-net) have been proposed as a way to reduce the dimensionality of genomic data and alleviate the issues associated with the small *n* large *p* problem. Daetwyler et al. [16] showed using a simulation scheme that BayesB [17] tends to have an advantage compared to GBLUP when the number of causal loci is less than the estimated number of independent chromosome segments.

In comparisons between GBLUP and BayesB using real data, the latter tends to yield superior results when the trait of interest is under the influence of at least one major gene, such as DGAT1 for fat and protein content in dairy cattle [18]. While BayesB tends to yield predictions that are at least as accurate as GBLUP in most practical analyses, it is computationally demanding, particularly as the number of predictors included in the model increases. Principal component analyses can dramatically reduce the dimensionality of the association model without a substantial loss in the portion of explained genetic variance; however, the estimated effects are linear combinations of the original predictors, thus complicating their interpretation. In general, the gains from using variable selection methods have been modest to nonexistent.

While the presence of causal variant genotypes in sequence information might be expected to give variable selection methods an advantage, this has not been supported by results from real data [12–14, 19], likely due to the high dimensionality of the models and high LD of the causal variants with large numbers of neighboring markers.

Preselection of variants prior to training of the model has been suggested both as an alternative and complement to variable selection methods. Heidaritabar et al. [13] preselected SNPs based on mutation type (e.g., synonymous, nonsynonymous, and non-coding) from a full set of approximately 4.6 million markers but found no appreciable gain in accuracy. Other studies have attempted to identify the most relevant variants through association statistics such as *p*-values, absolute estimated effects, or the relative contribution to the genetic variance. Investigating inbred lines of *D. melanogaster*, Ober et al. [11] selected the top 5% of SNPs ranked either by absolute estimated effect or the proportion of the genetic variance explained and found no significant improvement of accuracy using either preselection criteria. Veerkamp et al. [20] preselected variants based on *p*-values in Holstein data and found no improvement in accuracy, with the additional disadvantage of bias in the GEBVs and inflation of the variance component estimates. Frischknecht et al. [21] used *p*-values, annotation missense status, or LD-pruning to preselect variants; LD-pruning was the only strategy that did not reduce accuracies. Some studies have combined preselected SNPs with standard medium-density SNP chips to compromise between the potential benefits of each marker set, but few have found any benefit from this approach [22–25]. However, many of these studies performed SNP discovery and training of the prediction model using the same reference data set.

These results are not surprising and are in fact a consequence of the Beavis effect [26], a variation of the so-called “winner’s curse” phenomenon, where many of the selected SNP effects are overestimated, which will result in biased predictions and reduced accuracies in the validation set. Many studies that have investigated marker preselection based on association statistics criteria (e.g., *p*-values, absolute estimated effects) have used the data twice (in preselection and training), and this could be the primary explanation for their failure to improve accuracies. Splitting the data into three non-overlapping sets for discovery, training, and validation may alleviate this bias; however, this is a suboptimal use of an expensive resource and could result in an increase in the standard error of estimates and corresponding decrease in power to detect relevant markers. Additionally, splitting the data may not eliminate the population structure that arises from families or breeds, which can contribute to an erroneously inflated association of markers with the trait [27].

Toghiani et al. [28] introduced the population statistic F_ST_, a measure of deviation in allele frequencies between populations, as a criterion for marker preselection in genomic evaluations of livestock. They showed that by using high- and low-phenotype individuals within a population to calculate F_ST_ scores, historical selection signals could be detected at markers that tag causal loci. Chang et al. [6] demonstrated that preselection of markers by F_ST_ scores could significantly improve genomic prediction accuracies, and even outperformed BayesB and BayesC as the dimensionality of the model increased. A subsequent study by Chang et al. [7] showed that genomic similarity between individuals will be maximized using a highly stringent subset of the top markers as ranked by F_ST_ scores, though accuracies will not be maximized using this subset. They proposed that the highest potential accuracy will be achieved when a balance between high genomic similarity and the proportion of genetic variance explained is achieved.

In this study, we expanded upon these results by investigating how the inclusion of markers in linkage equilibrium with causal loci impact the estimation of genomic relationships and affect prediction accuracies. Additionally, we compared the sensitivity of F_ST_ scores and estimated SNP effects as preselection criteria to discriminate between markers that are linked and unlinked with causal loci and the potential of each to increase accuracies.

## Results

**Fig. 1:**
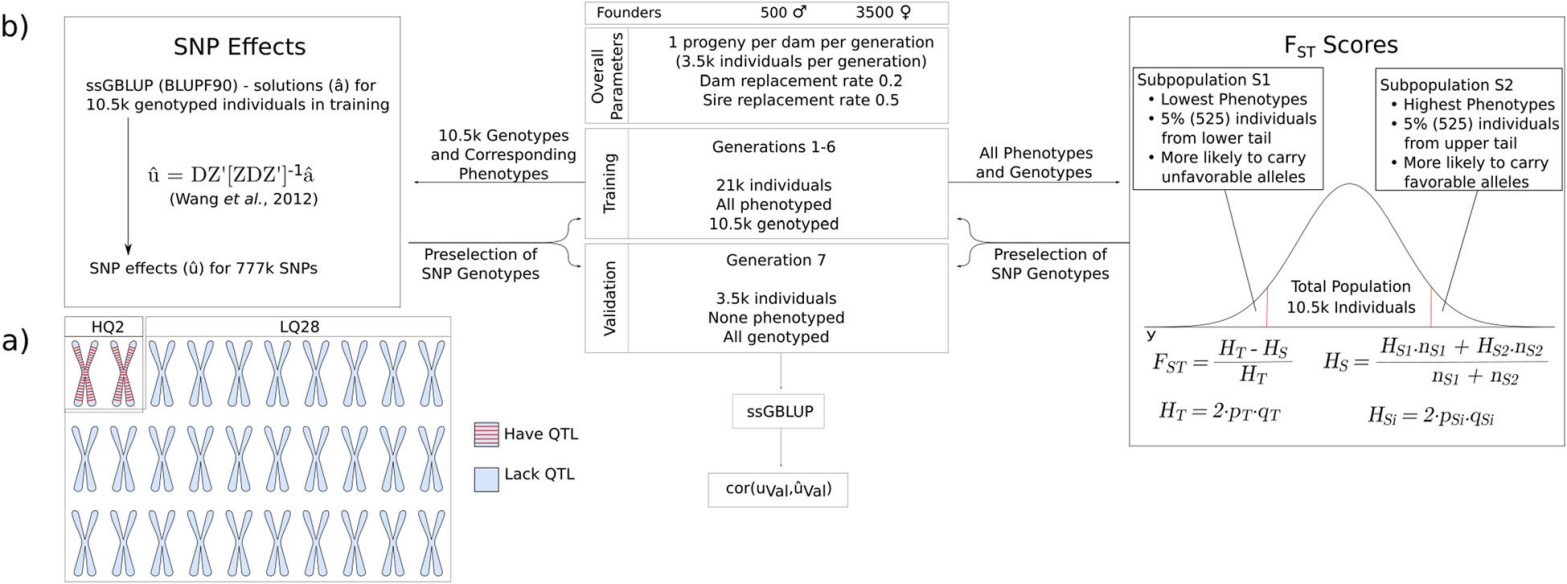
A general description of the simulation and workflow: **a**) A 30-chromosome genome was simulated with 200 QTL randomly distributed across 2 chromosomes and the remaining 28 chromosomes harboring no QTL. **b** A schematic representation of the pedigree simulation (7 generations of 3.5 k individual each). The first six generations (21 k phenotyped individuals and half of them genotyped) were used for training. The last generation consisting of 3.5 k genotyped and non-phenotyped individuals was used as validation set. Preselection of SNPs was based either on the absolute estimated marker effects or F_ST_ scores calculated using data from the training population

Accuracy of prediction was 0.37, 0.62, 0.89 and 0.22 using pedigree, all, HQ2, and LQ28 markers, respectively, to model the relationship matrix. As expected, the highest (0.89) and lowest (0.22) accuracies were obtained when the genomic relationship matrix was constructed using only linked (HQ2) or unlinked (LQ28) markers (Fig. 1a), respectively. Using the latter, accuracy was 39.6% lower than that achieved using expected relationships despite being based on genomic information. While use of all 777 k markers outperformed expected relationships by 70.3%, the accuracy was still approximately 30% lower than that obtained using only HQ2 SNPs.

Accuracies based on marker subsets preselected either randomly, by F_ST_ scores, or by estimated effects are shown in Table 1. When markers were preselected randomly, accuracy increased rapidly and plateaued when approximately 20 k markers were used. This is similar to the trend observed using commercial genotyping panels, where a subset of reasonably well-distributed markers yielded prediction accuracies similar to much higher density platforms. Although 50 to 60 k markers are typically necessary for many livestock species before reaching a plateau in accuracy, the smaller number of SNPs required in this study is likely due to the unconventional simulated genome structure and high LD between markers and QTL.

**Table 1:** Accuracy of genomic predictions under varying number of random-, F_ST_-, or estimated effect-based preselected markers

Selection method^a^	Number of preselected SNPs (in thousands)					
	1	10	20	30	40	50
Random	0.27	0.51	0.57	0.59	0.59	0.60
F_ST_	0.81	0.85	0.83	0.81	0.80	0.79
Effect	0.84	0.78	0.72	0.69	0.68	0.67

^a^ SNPs were preselected either randomly, based on their F_ST_ scores, or based on the absolute value of their estimated effect

Use of markers preselected based on F_ST_ scores resulted in a higher accuracy compared to the use of all markers. In fact, accuracy increased between 26.7 and 36.4% across all subsets. Accuracy peaked with the use of the top 10 k markers and remained fairly persistent; the decrease in accuracy was only 7.1% as the number of preselected markers increased to 50 k.

For preselection based on SNP effects, accuracy for 1 k markers was initially comparable to that achieved using 10 k F_ST_-preselected markers (0.84 and 0.85, respectively); however, accuracies rapidly declined (by 20.2%) with larger subsets and the top 50 k markers yielded accuracies that exceeded use of all markers by only 8%.

Table 2 shows the percentage of preselected markers that are located on either of the two chromosomes harboring QTL. These percentages are measures of the sensitivity of the preselection criteria to detect markers that are truly linked with causal loci. The top 1 k F_ST_-preselected markers were almost all (99.99%) SNPs in true linkage with QTL. The sensitivity steadily declined as the number of preselected markers increased and reached a minimum of only 28% linked when 50 k markers were preselected. Preselection by SNP effects followed a similar trend but had greater sensitivity to detect markers potentially linked with QTL for all subsets compared to F_ST_.

**Table 2:** Overlap (%) between random-, F_ST_-, or effect-preselected marker subsets and G_2_ SNPs

Selection method^a^	Number of preselected SNPs (in thousands)					
	1	10	20	30	40	50
Random	6.75	6.65	6.63	6.64	6.63	6.69
F_ST_	99.99	67.04	47.19	37.84	32.26	28.45
Effect	100.00	76.07	54.13	43.13	36.42	31.89

^a^ SNPs were preselected either randomly, based on their F_ST_ scores, or based on the absolute value of their estimated effect

The proportion of genetic variance explained by preselected marker subsets is shown in Table 3. The genetic variance contributed by a particular QTL was considered explained by a marker subset if at least one marker had an r^2^ greater than 0.9 with the QTL. As expected, preselection using a random selection criterion explained the least amount of the genetic variance. Preselection by F_ST_ and absolute estimated effects resulted in significantly more genetic variance explained, as much as 40 and 41%, respectively. Yet for neither criteria did maximization of genetic variance explained coincide with maximization of prediction accuracy, likely as a consequence of an increasing proportion of unlinked markers present in larger subsets (Table 2).

**Table 3:** Proportion of total GV^a^ explained by random, effect, and F_ST_-preselected markers

Selection method^b^	Number of preselected SNPs (in thousands)					
	1	10	20	30	40	50
Random	0.0041	0.018	0.082	0.11	0.10	0.14
F_ST_	0.31	0.38	0.39	0.39	0.39	0.40
Effect	0.33	0.40	0.41	0.41	0.41	0.41

^a^*GV* Genetic variance ^b^SNPs were preselected either randomly, based on their F_ST_ scores, or based on the absolute value of their estimated effect

Genomic information increased accuracy compared to pedigree by improving modeling of the MS. The effectiveness of a set of markers to capture QTL similarity and MS between individuals could be evaluated by assessing the correlation between marker- and QTL-based **G** matrices. The non-centered **G** matrix reflects the total QTL similarity while the centered **G** matrix (Eq. 1) will reflect the MS component only.

Correlations between the marker- and QTL-based **G** matrices for all, HQ2, or LQ28 markers are listed in Table 4. As expected, the non-centered correlations followed the same trend as that observed for the accuracies, with the maximum (0.63) and minimum (0.28) correlation obtained using only HQ2 and LQ28 markers, respectively. When **G** was centered by the expected relationships, the correlation for LQ28 markers was effectively zero. In contrast, using only linked markers to construct **G,** the correlation decreased by just 8.4% after adjusting for expected relationships.

**Table 4:** Correlations between centered and non-centered genomic relationships with QTL relationships for different sets of markers^a^

	All	HQ2	LQ28
Non-Centered	0.345399	0.631371	0.284554
Centered	0.159684	0.578165	0.0017988
Relative Decrease (%)	0.537721	0.084473	0.993687

^a^ All = all markers; HQ2 = markers on the two chromosomes harboring the QTL; LQ28 = markers on the 28 chromosomes lacking QTL

**Fig. 2:**
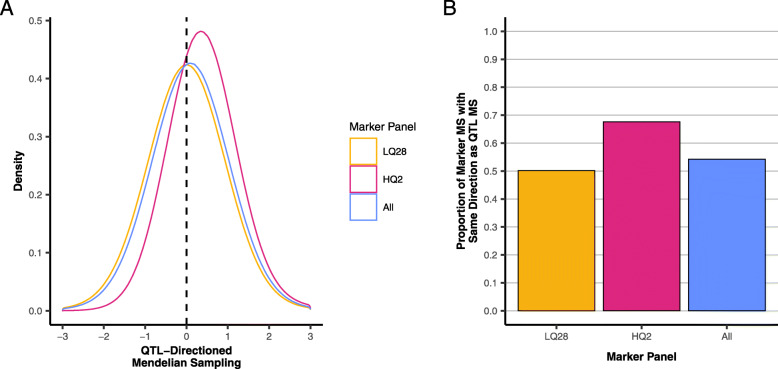
Characterization of the modelling of QTL Mendelian Sampling (MS) using all, HQ2, and LQ28 markers: **a**) The distribution of marker-estimated MS for relationships among training individuals with sign reflecting whether marker-estimated and QTL MS fall in the same (+) or opposite (−) direction relative to the expected additive relationship. **b** The proportion of relationships among training individuals for which marker-estimated and QTL MS fall in the same direction relative to expected additive relationships

This independence between the variation of LQ28 markers and QTL is illustrated in Fig. 2a, which plots the density of Eq. 2 for all, HQ2, and LQ28 markers. For the LQ28 subset, the distribution of this directional MS component falls evenly around zero; the number of marker-estimated relationships that fail to capture the correct direction of the QTL MS and the number that capture it correctly are approximately equal (Fig. 2b). The distribution for HQ2 is shifted towards more positive values, showing that this group of markers estimates the correct direction of the QTL MS more often than not. Interestingly, HQ2 markers still fail to capture the correct direction of the MS of QTL approximately 30% of the time (Fig. 2b); this likely occurs primarily when the deviation of the QTL genomic relationship from the expectation is quite small.

**Fig. 3:**
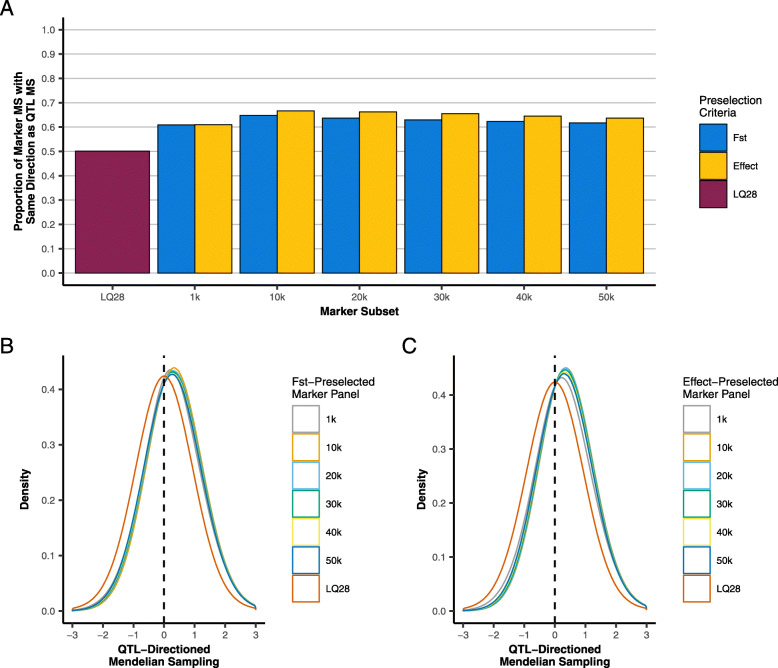
Characterization of the modelling of QTL Mendelian Sampling (MS) based on F_ST_- and estimated-effects-preselected markers: **a**) The proportion of relationships among training individuals for which marker-estimated and QTL MS fall in the same direction relative to expected additive relationships. **b** and **c** The distribution of marker-estimated MS for relationships among training individuals with sign reflecting whether marker-estimated and QTL MS fall in the same (+) or opposite (−) direction relative to the expected additive relationship

Tables 5 and 6 show the non-centered and centered correlations of the QTL-based **G** with **G** based on F_ST_- and effect-preselected subsets, respectively. For F_ST_, the correlation followed a similar trend as that observed for the accuracies (Table 1), with the largest correlation for both non-centered and centered **G** matrices achieved using the top 10 k F_ST_-preselected markers. The correlation for effect also peaked at the top 10 k markers, however, this does not coincide with where the accuracy is maximized. The relative decrease in the correlation with centering was smaller for SNP effects than for F_ST_-score-based prioritization, indicating that marker effects have a slightly better ability to capture the direction of the MS of QTL (Fig. 3a). However, both preselection criteria for all subsets considered were more likely than not to identify the true direction of the MS, as presented in Fig. 3b and c.

**Table 5:** Correlations between non-centered and centered genomic and QTL relationships for varying numbers of F_ST_-preselected markers

	Number of preselected SNPs (in thousands)					
	1	10	20	30	40	50
Non-Centered	0.339069	0.542457	0.54376	0.527988	0.511761	0.49678
Centered	0.315198	0.477285	0.469059	0.451059	0.433872	0.417522
Relative Decrease (%)	0.0728319	0.121576	0.138934	0.147398	0.153974	0.161264

**Table 6:** Correlations between non-centered and centered genomic and QTL relationships for varying numbers of estimated effects-preselected markers

	Number of preselected SNPs (in thousands)					
	1	10	20	30	40	50
Non-Centered	0.378834	0.607054	0.602191	0.576295	0.550798	0.529322
Centered	0.351288	0.550309	0.548288	0.528462	0.506049	0.48496
Relative Decrease (%)	0.0739304	0.093814	0.0899005	0.08346	0.0818111	0.0844371

**Fig. 4:**
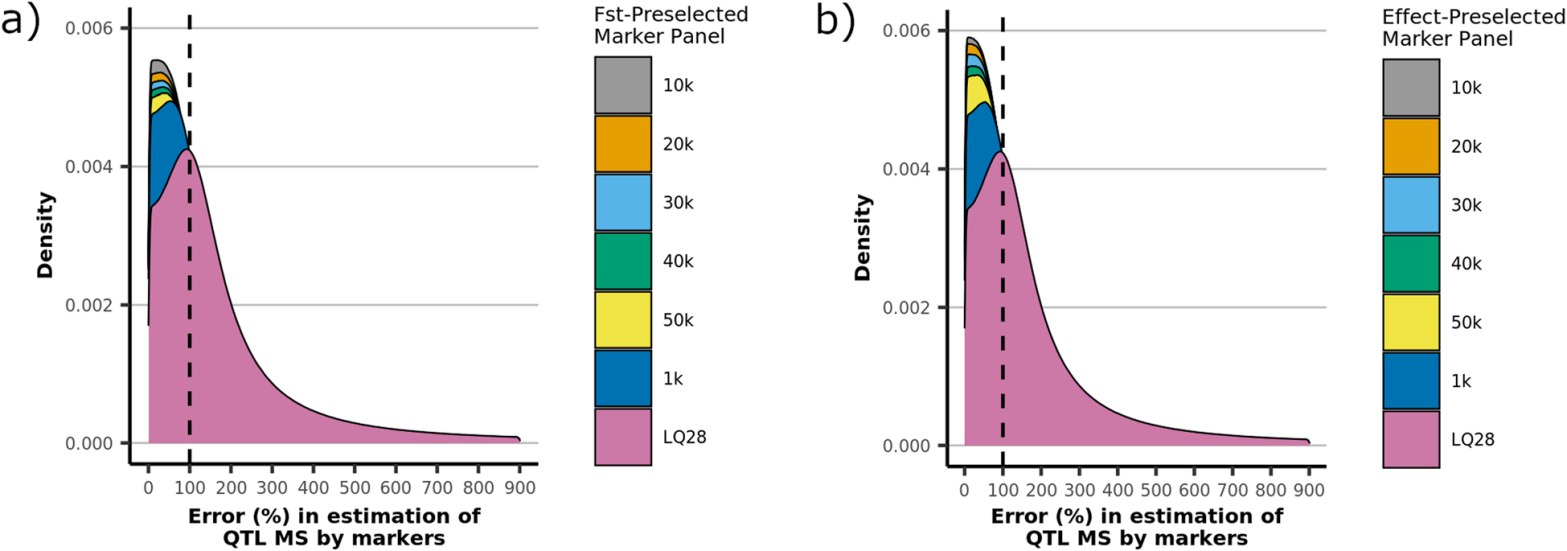
Errors in the estimation of QTL Mendelian Sampling: Distribution of error terms (%) in the estimation of genomic relationships (Eq. 3) for a) F_ST_ - and b) estimated effect-preselected marker subsets

Figure 4 presents the distribution of the errors in estimating the MS of the QTL (Eq. 3) using subsets of markers preselected by F_ST_ and absolute estimated effects. For both preselection methods, the error was minimized when only 10 k markers were preselected (highest density near zero). This coincides with the subset that maximizes accuracy for F_ST_, but not for preselection by estimated effects. Preselection based on the magnitude of the estimated effect maximized the accuracy using 1 k markers, which actually appears to yield the greatest error in MS estimation among the subsets considered.

**Fig. 5:**
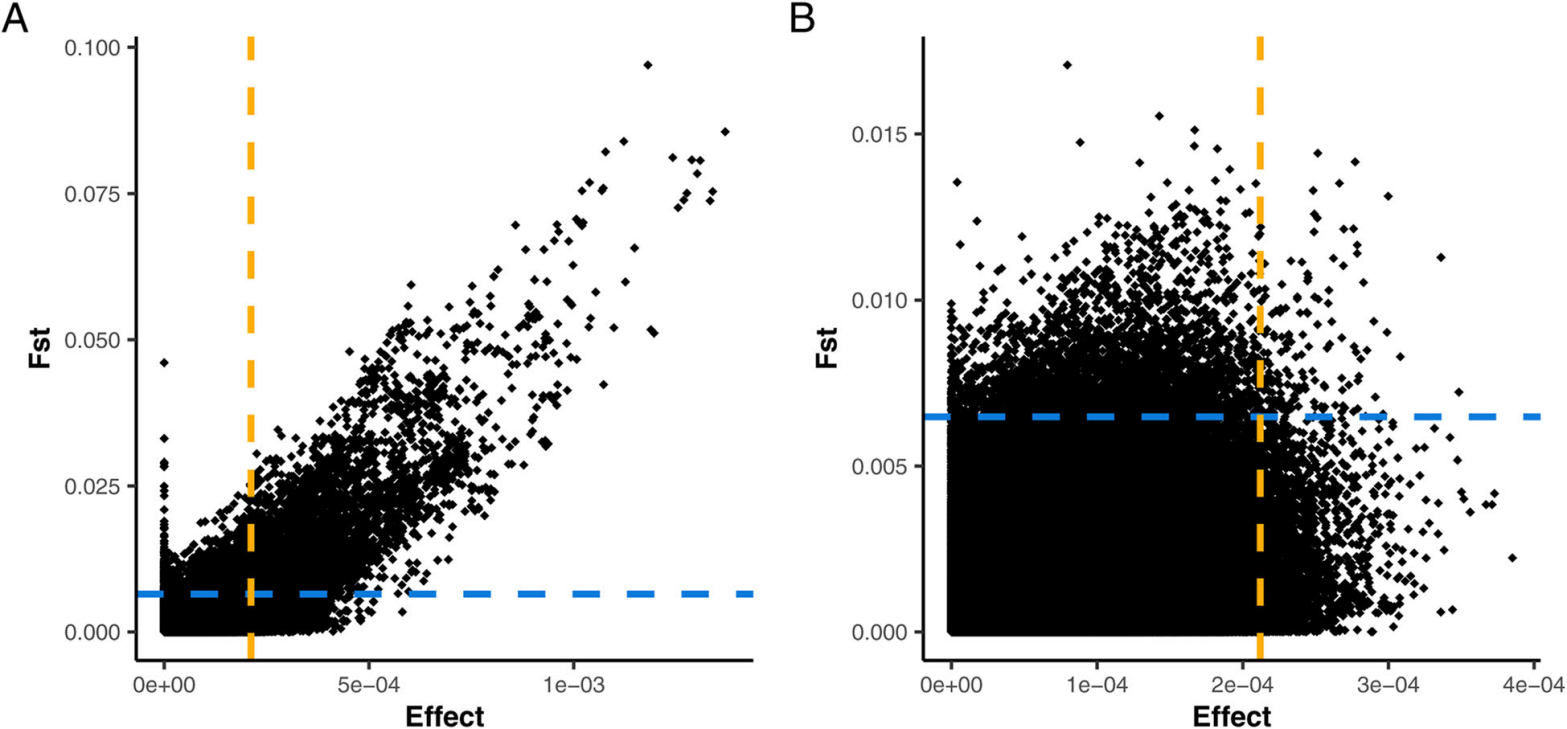
Regression of F_ST_ scores on the absolute estimated effect for **a**) HQ2 and **b**) LQ28 markers: The blue and yellow dashed lines denote the thresholds for selection of the top 10 k markers among all markers for F_ST_ and absolute estimated effects, respectively

When only 1 k SNPs were prioritized, the estimated effects preselection method seems to outperform the F_ST_-score-based approach. However, beyond the top 1 k panel, F_ST_ preselection consistently yields significantly higher accuracies. This coincides with when the sensitivity of both preselection methods starts to decrease, and unlinked markers begin to form part of the preselected subsets. This suggests that the difference between the two approaches is a consequence of the unlinked markers selected. Figure 5a and b show the regression of F_ST_ on estimated effect for HQ2 and LQ28 markers, respectively. There is a more consistent trend between the two statistics for HQ2 than for LQ28 markers. The Pearson correlation between F_ST_ and estimated effect is 0.77 and 0.27 for HQ2 and LQ28 markers, respectively. Together these results suggest that the two statistics tend to have high agreement when a prioritized marker is linked with a QTL but less so when the marker is unlinked.

In Fig. 5b, the threshold for inclusion in the top 10 k marker subsets for F_ST_ and estimated effects are denoted by a yellow and blue lines, respectively. It is clear that more SNPs with a large spurious association are preselected when using estimated SNP effects rather than F_ST_ scores. Without an independent training dataset, these large spurious associations will be re-estimated and exacerbated when training the prediction model and negatively affect the prediction accuracy in the validation set. The higher and more persistent accuracy for larger subsets when using F_ST_ as a preselection tool could be explained by its tendency to select markers that on average have less pronounced spurious associations.

To investigate this further, the top or bottom 50 k LQ28 (unlinked) markers as ranked based on F_ST_ scores or absolute estimated effects were excluded from the full panel of 777 k SNP markers. The reduced panels of 725 k markers were then used for predictions and the resulting accuracies are presented in Table 7. Theoretically, given their lack of linkage with any QTL, it is expected that the excluded 50 k top or bottom markers should not influence the accuracy. However, that was not the case and exclusion of certain unlinked markers yielded an increase in accuracy, indicating that the analysis benefits from their absence.

**Table 7:** Accuracy after exclusion of different subsets of LQ28 markers from construction of the genomic relationship matrix

	Excluded markers^b^		
Exclusion criteria^a^	None	Top 50 k	Bottom 50 k
Effects	0.62	0.68	0.62
F_ST_ Scores	0.62	0.65	0.63

^a^ Markers were excluded from the LQ28 subset based either of their F_ST_ scores or effects; ^b^ All markers were included (None), top 50 k markers excluded (Top 50 k), and bottom 50 k markers excluded (Bottom 50 k)

Exclusion of the 50 k unlinked markers with the largest estimated effects resulted in the largest increase in accuracy (approximately 8.6%) compared to use of all markers without preselection. In contrast, exclusion of the 50 k unlinked markers with the smallest estimated effect led to no change in accuracy relative to use of all markers, as expected given that their estimated effects were close to zero. However, exclusion of the 50 k unlinked markers with the largest F_ST_ scores resulted in a smaller increase in accuracy (4.1%), showing the superiority of the F_ST_ method in avoiding the preselection of unlinked markers with pronounced spurious associations.

While the simulation design previously evaluated is convenient for evaluating the behavior of markers that are unlinked with QTL in a prediction model, it would be unreasonable to expect a complex trait in reality to be accurately modeled by such a design. To evaluate whether a similar trend could persist under a more reasonable distribution of QTL across the entire genome, the simulation was repeated with the 200 QTL distributed across all 30 chromosomes. Table 8 shows accuracy and percentage of genetic variance explained for F_ST_- and effect-preselected subsets.

**Table 8:** Accuracy and percent of genetic variance explained by F_ST_- and effect-preselected subsets under a simulation design with 200 QTL distributed across all 30 chromosomes

		Number of preselected SNPs (in thousands)					
		1	10	20	30	40	50
Accuracy	F_ST_	0.66	0.73	0.73	0.72	0.71	0.71
	Effect	0.73	0.70	0.67	0.66	0.65	0.64
GV Explained (%)	F_ST_	0.21	0.29	0.31	0.32	0.32	0.33
	Effect	0.21	0.34	0.35	0.35	0.35	0.36

With QTL distributed across all chromosomes, accuracy using all markers was 0.60. Both preselection methods achieve a maximum accuracy of 0.73, though F_ST_ requires a larger number of preselected markers to achieve this. As the panel size increases to 50 k, the accuracy for effect- and F_ST_-preselection decrease by approximately 12.3 and 2.7%, respectively. Despite yielding a lower accuracy for panels of 10 k markers and larger, the effect-preselected subsets explain 9.1 to 17.2% more of the genetic variance than the equivalently-sized F_ST_-preselected subsets. This demonstrates that the trend in prediction results for F_ST_- and effect-preselected subsets is consistent even when all chromosomes harbor multiple causal loci.

## Discussion

It was shown that the predictive ability of markers that are unlinked with QTL is inferior to even pedigree information, a result that agrees with previous studies [29–31]. However, despite their inferior predictive power, accuracies using only unlinked markers were always positive. Habier et al. [29] attributes this to unlinked markers modeling additive genetic relationships and shows that the accuracy will converge to that of pedigree BLUP as the number of independently segregating markers increases. Regardless of linkage, the distribution of QTL and marker additive relationships for a particular order of kinship will share a mean, the expected relationship. The advantage of using genomic information compared to pedigree is the better modeling of the MS of QTL. However, when markers and QTL segregate independently the covariance of marker and QTL MS is zero (Table 4) and the marker-based relationships are noisy estimates of the average additive relationships. While these markers will independently yield positive accuracies, they should not be expected to benefit the analysis when markers in LD with causal loci are available. HQ2 markers also capture the additive relationship with the additional benefit of accounting for some portion of the MS of QTL, as evidenced by the limited decrease in the correlation between the HQ2-marker- and QTL-based **G** matrices after centering with expected relationships (Table 4) and the shift of the HQ2 distribution in Fig. 2a to more positive values.

Ideally, the effect of unlinked markers on the estimation of the breeding values would be zero when more informative markers are present in the model. However, the inferior accuracy obtained using all markers compared to only HQ2 markers demonstrates that the effect of unlinked markers will not be null. The results of this study demonstrate that in terms of a GBLUP model, allowing unlinked markers to have a nonzero contribution to **G** adds noise to the estimation of genomic relationships that will not be reflective of true QTL similarity, resulting in lower accuracy relative to that achieved using only linked markers in the validation population. In terms of a SNP-BLUP model, which has been shown to be equivalent to GBLUP [29], nonzero estimates will be obtained for unlinked markers that have no association with QTL inheritance in validation individuals. Table 4 shows that the MS of QTL and unlinked markers vary around the same average relationship, which creates an association of the unlinked markers with the QTL. The model cannot discriminate spurious marker associations that are a result of this shared expectation and random sampling from associations due to true linkage with a causal locus, particularly when the unlinked markers are themselves used to inform the variance-covariance structure.

These results highlight the motivation and potential for preselection of markers to improve accuracies. Both F_ST_ scores and absolute estimated effect preselection-based methods were able to identify relevant markers with high sensitivity when preselecting a small number of markers and yielded high accuracies. However, the trend in accuracy differed substantially between the two approaches. As the number of preselected markers increased, their sensitivity to detect linked markers decayed, and unlinked markers were incorrectly selected. Preselection by F_ST_ increased accuracy from 1 k to 10 k markers while the accuracy for preselection by estimated effects decreased by approximately 7.1% over the same interval. This occurred despite F_ST_ preselection adding 903 more unlinked markers and explaining approximately 5% less of the genetic variance than estimated effects. The accuracy for F_ST_ preselection declined as the number of preselected markers increased beyond 10 k, but was more persistent than the accuracy for estimated effects despite consistently selecting more unlinked markers and explaining less of the genetic variance.

There are two important concepts that are illustrated by the behavior of these statistics. First, when the preselection criteria have imperfect sensitivity, accuracy will be maximized by a balance between increasing the genetic variance explained and minimizing deleterious contributions from poorly informative markers. F_ST_ added a large number of unlinked markers when the number of preselected SNPs increased from 1 k to 10 k, but the genetic variance explained was also significantly increased, resulting in an overall improvement in accuracy. As long as the beneficial contribution to the genetic variance explained by linked markers exceeds the negative effects of the association noise added by unlinked markers, the accuracy will increase. The decline in accuracy for F_ST_ when the number of preselected markers increased from 10 k to 20 k is explained by the fact that the genetic variance explained increased by only 2.6% while approximately 73% of added markers were unlinked with QTL; this likely contributed significant noise to estimation of genomic relationships. This is in concordance with Chang et al. [7], who concluded that a balance is needed between genomic similarity and the proportion of genetic variance explained by the preselected markers in order to maximize accuracies. While in the current study we make only a distinction between linked and unlinked markers, markers that are linked to but in low LD with a QTL will also contribute noise to the model and the negative impact of this noise may outweigh the benefit of any genetic variance they explain.

Second, the noise contributed by unlinked markers is not necessarily equal between both preselection methods. Estimated-effects-based approach consistently showed a greater sensitivity to detect linked markers than F_ST_, yet yielded significantly lower accuracies, except in the case of the 1 k panel where it selected no unlinked markers. For panel sizes of 10 k and larger, the accuracy for the estimated-effects-based approach was lower than for F_ST_ scores largely because the unlinked markers selected by the approach have a greater detrimental effect.

When the 50 k most spuriously associated unlinked markers were excluded from the analysis (Table 7), accuracies improved significantly. These markers have a large spurious association with the trait and the analysis benefits from their exclusion. While the complications that such markers present are often considered in the context of marker preselection, this result shows that such markers will have an appreciable negative impact even in the absence of preselection. There is therefore an incentive to identify and filter spuriously associated markers if a reliable and efficient method for distinguishing them from true associations can be developed.

Excluding the 50 k LQ28 markers with the largest F_ST_ scores from the full panel also resulted in the accuracy increasing, but this increase was not as pronounced as when the LQ28 markers with largest estimated effect were excluded. This indicates that when the training data is also used to calculate F_ST_ for preselection, there will be some tendency to select irrelevant markers with a spurious association, but that the spurious associations will on average be less severe than when preselecting by the absolute estimated effects. This could explain why accuracies are more persistent for preselection by F_ST_ scores than estimated marker effects even when the F_ST_ preselection criteria selects more unlinked markers and explains less of the genetic variance.

Both F_ST_ and marker effects were estimated using some portion of the training data rather than an independent dataset. While partitioning of the training data into two subsets, one for estimation of preselection statistics and one for training of the prediction model, may alleviate some bias, it will decrease the size of the data available for training the model and therefore increase the standard error in estimation of the statistics anyway. Splitting of the training data will not be a feasible option for most analyses, and the literature shows that several analyses that consider preselection by association statistics in genetic improvement programs have chosen to reuse the SNP discovery data for training of the model.

In contrast to marker effect estimation, calculation of F_ST_ used just 10% of the training data (Fig. 1). Spurious associations present in the full training data may be less extreme in subsets of that data, which could explain why F_ST_ is less affected by the bias that results from using the same data for both preselection and model training. F_ST_ then has the potential to be a simple and efficient preselection tool that can reduce the bias associated with preselection by association statistics without requiring an inefficient partitioning of the training data or expensive collection of new independent data.

F_ST_ scores and association statistics could potentially be combined into an index to harness the benefits of both preselection statistics. The Pearson correlation between F_ST_ scores and estimated effects was 0.78 and 0.28 for HQ2 and LQ28 markers, respectively. This suggests that there is high agreement among the two statistics when markers are linked with QTL, but much less so among unlinked markers. Spuriously associated markers could possibly be identified and excluded when there is large disagreement between the two statistics.

An additional benefit of F_ST_-based prioritization is that it is not affected by an increase in the number of markers included in the model due to the independence in calculating the score of each marker. As the number of markers in the association model increases, estimation variance for estimated effects of markers will increase without a corresponding increase in the size of the training data set. Furthermore, the estimated effect of each marker will be further regressed toward zero as QTL effects become distributed over correlated blocks of the predictors [32]. This will further complicate disentangling true from spurious associations as both take a similar magnitude of estimated effect. In contrast, F_ST_ scores will remain constant regardless of the number of markers, correlated or uncorrelated, that enter jointly into the analysis. This does carry the drawback that highly correlated markers will have similar F_ST_ scores and so selecting only by top F_ST_ score will select all correlated markers in a block, which could cause bias [21] and inflation of variance estimates [20] due to multicollinearity. While not evaluated in this study, these issues could be avoided through LD-pruning of F_ST_-selected markers or similar filtering measures.

Variable selection models are a conceptually similar but fundamentally different approach to marker preselection for reduction of the parameter space. While we do not explore a comparison of F_ST_ and variable selection models in this study, Chang et al. [6] compared F_ST_ preselection implemented in a BayesA-like regression with BayesB and BayesC. They found that while F_ST_ preselection did not outperform the Bayesian variable selection models in all scenarios, it did tend to have an advantage as the density of the full panel increased. In general, they found that BayesB and BayesC accuracies decreased with increased density of available markers, while accuracy using F_ST_ preselection tended to improve. This seems to be a result of the decreased statistical power to identify relevant markers flanking QTL of low effect as the number of parameters in the model increases. The benefits of both approaches might be harnessed by using F_ST_ to preselect markers with a generous threshold followed by implementation in a variable selection model that includes the prioritized markers.

## Conclusions

In this study, F_ST_ was shown to be an efficient criterion for preselection of trait-relevant markers that can improve modeling of QTL similarity between individuals, increase prediction accuracies, and maintain more stable prediction accuracies than comparative association statistics like absolute estimated marker effects. While association statistics are powerful tools for identifying loci associated with a particular trait, disentangling spurious associations from weak but true signals is often not possible within the constraints of the data available. We showed that the more persistent prediction accuracy using F_ST_-score-prioritized markers was the result of the ability of the F_ST_-score-based method to select unlinked markers with weaker spurious associations with the trait compared to preselection based on absolute estimated effects. While this study only explored F_ST_ scores as an independent preselection statistic, we showed that F_ST_ and absolute estimated effect approaches preselected highly correlated sets of markers linked with QTL but significantly less so among unlinked markers. This highlights the potential for the possibility of combining F_ST_ scores (or similar population statistics) and association statistics into a powerful preselection index to reduce inclusion of nonrelevant markers with a large spurious association.

## Methods

### Data simulation

The simulated genome consisted of 30 chromosomes (100 centiMorgans each in length) that harbored a total of 777 k evenly-distributed SNP markers and was generated using QMSim [33]. As one of the primary goals of this study was to investigate how markers that segregate independently from QTL affect prediction accuracies, 200 QTL were randomly distributed across 2 of the 30 chromosomes, as illustrated in Fig. 1a. While it is an extreme scenario for real distribution of QTL for a complex trait, this design allowed unambiguous classification of approximately 725 k markers as segregating independently from any QTL, with the approximately 52 k remaining markers potentially linked with at least one QTL. QTL were limited to 2 chromosomes to ensure a high LD of the majority of markers on these chromosomes with at least one QTL and to evaluate the behavior of markers that are unlinked with QTL in a prediction model. QTL effects were sampled from a Gamma distribution with a shape parameter of 0.4.

The LD structure was generated through 2070 generations of random mating in a historical population, with a bottleneck occurring at generation 2000. The number of individuals in the historical population varied between 600 and 4000. The resulting average LD (r^2^) was 0.32 and 0.084 between consecutive SNP and QTL, respectively.

A trait with a heritability equal to 0.4 was simulated. The genetic and residual variances were set equal to 0.4 and 0.6, respectively. The model used to simulate the phenotypes included the true breeding value (the cross product of true QTL effects and genotypes) and a normally distributed error term with mean zero and dispersion equal to the residual variance.

All individuals from the last generation of the historical population (500 males and 3500 females) were selected to be founders of a population under selection (SP). An additional seven generations (3500 progeny each) were generated. Pedigree-based estimates of breeding values (pEBVs) were used for selection. Sire and dam replacement rates were set equal to 0.5 and 0.2, respectively. The training population consisted of the first six SP generations. It included 21 k phenotyped individuals; a randomly selected half of these were also genotyped. The seventh generation of SP consisted of 3.5 k genotyped individuals, none of which were phenotyped, and was used as the validation population. Ten replicates of the simulated genome and phenotypic data were generated.

### Methods

Predictions were made using a single-step GBLUP model (ssGBLUP) [34–36] as implemented in the BLUPF90 software [37]. The genomic relationship matrix (**G**) was constructed according to VanRaden [38],

$$ \boldsymbol{G}=\frac{\boldsymbol{ZZ}^{\prime }}{2{\sum}_{i=1}^n{p}_i\left(1-{p}_i\right)}, $$

Where **Z** is a matrix of SNP genotypes, *p*_*i*_ is the minor allele frequency of the *i*^th^ SNP, and *n* is the number of SNPs.

In order to evaluate the consequences that SNPs unlinked with QTL have on prediction accuracies, three analyses that differed in the linkage status of the SNPs used to build **G** were performed. The three SNP subsets considered were 1) only the 51,800 SNP situated on either of the two chromosomes harboring around 100 QTL each (HQ2), 2) only the 725,200 SNP on any of the 28 chromosomes that lack QTL (LQ28) and can be definitively classified as being unlinked with QTL, and 3) the union of the two previous subsets that includes all 777,000 SNP regardless of linkage status.

In the next stage of the study, F_ST_ was used as a criterion for preselection of SNP subsets and was compared to preselection according to absolute estimated marker effect or random subsets. Subsets of 1, 10, 20, 30, 40, and 50 k SNPs for each preselection criteria were used for the construction of **G** and corresponding prediction accuracies, defined as the Pearson correlation between true and estimated breeding values of validation individuals, compared.

F_st_ scores were calculated following Nei [39],

$$ {F}_{ST}=\frac{H_T-{H}_S}{H_T}, $$

where *H*_*T*_ = 2 ∙ *p*_*T*_ ∙ *q*_*T*_, ${H}_S=\frac{H_{S1}\bullet {n}_{S1}+{H}_{S2}\bullet {n}_{S2}}{n_{S1}+{n}_{S2}}$ and *H*_*Si*_ = 2 ∙ *p*_*Si*_ ∙ *q*_*Si*_,

where *p*_*T*_ and *q*_*T*_ are major and minor allele frequencies, respectively, in the population; *p*_*Si*_ and *q*_*Si*_ are major and minor allele frequencies, respectively, in the *i*^th^ subpopulation; and *n*_*Si*_ is the number of individuals in the *i*^th^ subpopulation. The genotyped and phenotyped individuals in the training population were ranked according to their phenotype and the bottom and top 5% of individuals used to create two subpopulations (S_1_ and S_2_), as illustrated in Fig. 1b. Using these subpopulations, an F_st_ score for each SNP was computed as indicated in formulae above. SNPs were then ranked based on their F_st_ scores and subsets of the top 1, 10, 20, 30, 40, and 50 k markers used to compute **G**.

The rationale for forming subpopulations from individuals of extreme phenotype in a single breeding population rather than using separate breeding populations with highly divergent phenotypes (e.g., milk production in Holstein-Friesian and Jersey) is to minimize the likelihood of preselection of adaptive SNP markers that are specific to a population due to natural or artificial selection. By obtaining extreme phenotypes from within a single breeding population, any potential divergence in allele frequency related to traits uncorrelated with the one of interest will be more effectively averaged over.

Estimated genomic breeding values that were obtained using all 777 k markers to compute **G** were used to derive SNP effects through the following relationship [40],

$$ \hat{\boldsymbol{u}}=\boldsymbol{DZ}^{\prime }{\left[\boldsymbol{ZDZ}\prime \right]}^{-1}\hat{\boldsymbol{a}}, $$

where **û** is the vector of SNP effects, **â** is the vector of estimated genomic breeding values for individuals in the training population with **G** modeled using all 777 k SNPs, **Z** is a known incidence matrix of SNP genotypes, and **D** is a diagonal matrix of weights. In this study, ***D*** was set equal to the identity matrix to convey equal weight to all SNPs. Estimation of genomic breeding values and the back-calculation of SNP effects were obtained using ssGBLUP [34–36] as implemented in BLUPF90 [37] and PreGSF90 [41], respectively.

Random SNP subsets were generated by random sampling from all available SNPs with no restrictions placed on the number of markers sampled from a particular chromosome or the proportion that were linked and unlinked with QTL. A generalized outline of the approach to the analysis for Fst and SNP effect preselection and predictions is summarized in Fig. 1b.

### Analysis of marker and QTL similarity

Genomic information improves prediction accuracies compared to pedigree primarily through a better modeling of the MS of QTL. To dissect how the various marker-estimated genomic relationships capture the true QTL similarity between individuals and how they contribute to the maximization of prediction accuracy, several metrics were used to quantify the agreement between marker- and QTL-based **G** matrices.

First, a correlation was calculated between all elements of the full marker- and QTL-based **G** matrices, as suggested by VanRaden [38]; this correlation reflects the adequacy of the estimated genomic relationships to capture both the expected relatedness and MS. To further evaluate how well each set of markers models the MS component specifically, expected relationships were subtracted from each genomic relationship, and a correlation between the resulting centered **G** matrices was calculated,

$$ cor\left({\boldsymbol{G}}_M-{\boldsymbol{A}}_{22},{\boldsymbol{G}}_{QTL}-{\boldsymbol{A}}_{22}\right). $$

where **G**_M_ and **G**_QTL_ are the marker and QTL relationship matrices, respectively, and **A**_22_ is the matrix of expected relationships based on pedigree information for genotyped individuals. It is possible that certain markers will capture variation in MS that is not consistent with the MS of QTL when LD between markers and QTL is low. If the marker-estimated and QTL relationships fall in opposite directions around the expected relationship, then the expected relationship will in fact be a better estimate than the marker-estimated genomic relationship. The ability of marker subsets to capture the correct direction of the MS of QTL was determined as,

$$ \boldsymbol{Directional}\ \boldsymbol{MS}=\left\{\begin{array}{c}\left|\frac{{\boldsymbol{G}}_M-{\boldsymbol{A}}_{\mathbf{22}}}{sd\left({\boldsymbol{G}}_{\boldsymbol{M}}\right)}\right|\  if\ same\ direction\  as\  QTL\  MS\\ {}-\left|\frac{{\boldsymbol{G}}_M-{\boldsymbol{A}}_{\mathbf{22}}}{sd\left({\boldsymbol{G}}_M\right)}\right|\  if\ opposite\ direction\ from\  QTL\  MS\end{array}\right. $$

where sd (**G**_M_) is the standard deviation over all relationships within **G**_M_, and the sign reflects whether the MS component for marker-estimated relationships falls in the same (positive) or opposite (negative) direction as the QTL MS relative to the expected relationship.

At a minimum, marker-estimated relationships should capture the correct direction of the MS of QTL in order to improve the modeling of relationships relative to the expectation. An ideal set of markers will additionally minimize the distance between marker- and QTL-based relationships. This distance can be approximated using the following formulae,

$$ \boldsymbol{MS}\ \boldsymbol{Error}\ \left(\%\right)=\left|\frac{\frac{{\boldsymbol{G}}_M-{\boldsymbol{A}}_{\mathbf{22}}}{sd\left({\boldsymbol{G}}_M\right)}-\frac{{\boldsymbol{G}}_{QTL}-{\boldsymbol{A}}_{\mathbf{22}}}{sd\left({\boldsymbol{G}}_{QTL}\right)}}{\frac{{\boldsymbol{G}}_{QTL}-{\boldsymbol{A}}_{22}}{sd\left({\boldsymbol{G}}_{QTL}\right)}}\right|x100\%. $$

This puts the discrepancy between marker-estimated and QTL relationships in terms of an error (%) relative to the scale of the MS of QTL. The closer a value is to zero, the less the discrepancy between the marker-estimated and QTL relationship. A value less than one implies that the marker-estimated relationship is a closer approximation of the QTL relationship than the expected relationship, while a value greater than one implies either that the marker-estimated relationship captures the correct direction but overestimates the QTL MS, or that the marker-estimated relationship has opposite direction of MS than the QTL. Figures were generated using the tidyverse package in R [42].

## Data Availability

The datasets used and/or analyzed during the current study are available from the corresponding author on reasonable request.

## Abbreviations

GS: Genomic selection
MS: Mendelian sampling
SNP: Single nucleotide polymorphism
LD: Linkage disequilibrium
QTL: Quantitative trait loci
SP: Population under selection
pEBV: Pedigree-based estimated breeding value
ssGBLUP: Single-step GBLUP
G: genomic relationship matrix
S_1_ and S_2_: Subpopulations 1 and 2
HQ2: Subset of markers linked with QTL
LQ28: Subset of markers unlinked with QTL
